# Tunable meta-device for large depth of field quantitative phase imaging

**DOI:** 10.1515/nanoph-2024-0661

**Published:** 2025-02-17

**Authors:** Jialuo Cheng, Zihan Geng, Yin Zhou, Zhendong Luo, Xiaoyuan Liu, Yinuo Xiang, Junxiao Zhou, Mu Ku Chen

**Affiliations:** Department of Electrical Engineering, 53025 City University of Hong Kong, Kowloon, Hong Kong SAR 999077, China; Institute of Data and Information, Tsinghua Shenzhen International Graduate School, Tsinghua University, Shenzhen, Guangdong 518071, China; State Key Laboratory of Terahertz and Millimeter Waves, City University of Hong Kong, Kowloon, Hong Kong SAR 999077, China

**Keywords:** meta-device, meta-lens, bifocal, polarization manipulation, quantitative phase imaging

## Abstract

In traditional optical imaging, image sensors only record intensity information, and phase information of transparent samples such as cells and semiconductor materials is hard to obtain. Quantitative phase imaging techniques are crucial for obtaining detailed phase information, but current methods often require complex interferometric setups or mechanical adjustments, limiting their practical applicability. Here, we proposed a novel meta-device integrating a PB phase-based meta-lens, a refractive lens, and an electronically tunable lens with a polarization camera to capture multiple defocused images simultaneously for the transport of intensity equation-based phase retrieval algorithm. By leveraging the distinct focus lengths for left-circularly polarized and right-circularly polarized light, the meta-device eliminates the need for multiple shots and mechanical movements. Our approach enables rapid, precise, quantitative phase imaging at different depths. The experiment shows the accuracy of our methods is 98.47 % and with a 2.52 mm depth range of the objects that can be retrieved, making it highly suitable for dynamic and depth-varying samples, such as cells in solution.

## 1 Introduction

Traditional image sensors are limited to recording only the intensity profile of incident light, thereby losing all information about the phase distribution [1]. This limitation arises from the oscillation frequencies of light being significantly higher than the response speed of sensors. In scientific research, many of the samples of interest are transparent in terms of intensity and primarily possess the ability to modulate phases, such as cells, microlenses, and semiconductor materials. Consequently, obtaining phase information about these samples is of paramount importance. Phase imaging techniques can be broadly categorized into qualitative phase imaging and quantitative phase imaging (QPI). Qualitative phase imaging methods, such as Zernike phase contrast [2] and differential interference contrast (DIC) [3], typically provide only a rough estimate of the phase information, which aids in visual observation but lacks the specificity required for further research analysis.

QPI has emerged as a pivotal methodology in recent years, attracting substantial attention due to its ability to acquire comprehensive phase information, significantly facilitating subsequent analytical processes. QPI techniques can be further divided into interferometric and non-interferometric methods. Interferometric methods offer relatively high precision but are constrained by the stringent requirements for highly coherent light sources and stable optical setups, making them more complex and susceptible to noise [4], [5]. Among the non-interferometric methods, phase retrieval has seen extensive research in recent years. This includes approaches based on the Gerchberg–Saxton (GS) iterative algorithm [6], [7], which recovers the phase by using multiple-intensity images captured under different conditions, such as different distances [8], [9], [10] and different incident lights [11], [12], [13], [14]. One of the deterministic algorithms is the transport of intensity equation (TIE) [15]. TIE can operate under partially coherent illumination, offering high resolution, with a simple experimental setup and rapid phase calculation when using fast-Fourier transform (FFT)-based methods [16], [17]. However, the application of TIE is often limited by the need to mechanically move the object or sensor to acquire intensity images at different distances.

Meta-devices, composed of sub-wavelength artificial nanostructures, offer advanced wavefront modulation capabilities [18], [19], [20], [21], [22], [23], [24], [25], [26], [27], [28]. Several studies explore the use of meta-devices for QPI [29], [30], [31], with most existing works relying on interferometric methods. Fewer studies work with partially coherent [25], leveraging chromatic aberration of meta-lens [32], [33] or dual-focus meta-lenses [34]. These approaches enable the acquisition of multiple defocused intensity images without altering the position of the object or sensor, facilitating phase recovery using TIE.

However, it is still a practical limitation to performing QPI for objects at varying depths without adjusting the position of objects or sensors. This constraint is particularly problematic when observing cells within a solution of a certain thickness. Adjustable optical imaging technology plays an important role in this demand. Recent advancements have sought to mitigate the need for mechanical movement, such as using chromatic aberrations [32], spatial light modulators [35], volume holography [36], and electronic tunable lenses [37]. Here, we introduce a meta-device comprising a Pancharatnam–Berry (PB) phase-based meta-lens, a refractive lens, and an electronically tunable lens (ETL) integrated with a polarization camera. The meta-device is designed to exhibit distinct focus depths for left-circularly polarized (LCP) and right-circularly polarized (RCP) light, enabling the simultaneous acquisition of two defocused images on the polarization camera for TIE phase retrieval. The incorporation of the ETL allows for the simultaneous adjustment of the focal lengths for both LCP and RCP light, thereby facilitating QPI at varying depths. Using a light-emitting diode (LED) as the light source provides partially coherent illumination, enhancing imaging resolution while reducing the impact of environmental noise compared to traditional interferometric-based QPI methods. In contrast to conventional TIE algorithms that rely on mechanical movement to capture defocused images of an object’s front and back surfaces, our meta-device features two independent focal points capable of simultaneous imaging of these surfaces. Traditional TIE algorithms are limited in their ability to image objects at varying depths, whereas the meta-device can simultaneously electronically adjust the focal lengths to image objects at different depths, significantly broadening the application scope of TIE under LED illumination.

## 2 Results

**Figure 1:**
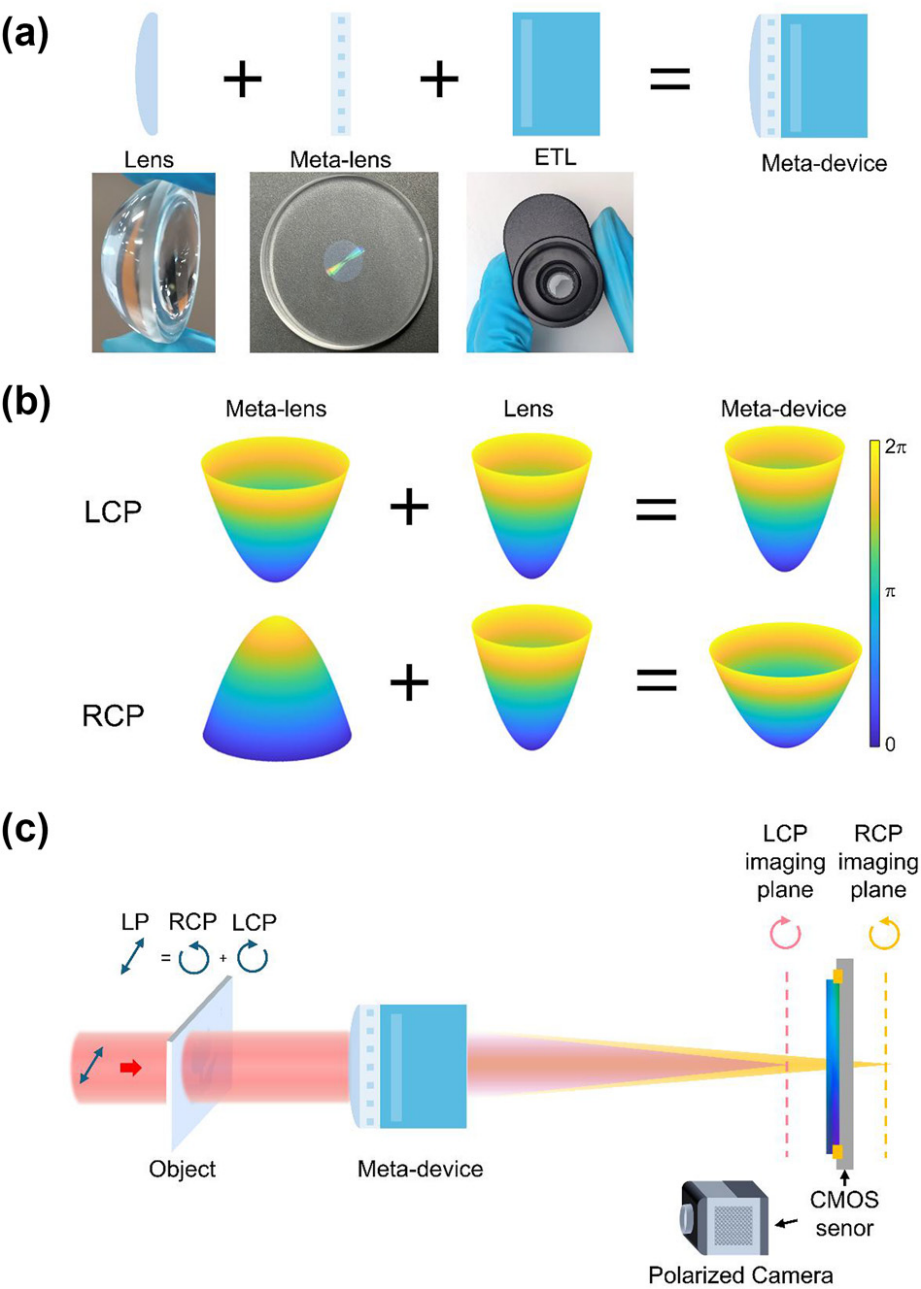
The diagram of the tunable meta-device. (a) The meta-device comprises a PB phase meta-lens, a refractive lens, and an ETL. (b) The phase distribution of introduced by the meta-lens, the refractive lens, and the meta-device from left to right. The first and second rows refer to LCP and RCP, respectively. (c) Linearly polarized (LP) light illuminates the meta-device. It spatially separates the LP light into LCP and RCP light at distinct locations along the propagation direction. A polarized camera positioned at the center between the two imaging planes captures simultaneous underfocused and overfocused images.

Figure 1 shows the concept of this tunable meta-device, which is designed to capture simultaneous underfocused and overfocused images using a polarized camera. The meta-device is composed of a PB phase-based meta-lens, a refractive lens, and an ETL, as depicted in Figure 1(a). The meta-lens is constructed from unit cells with varying rotation angles $\theta \left(x,y\right)$ which correspond to different phase modulations $\varphi \left(x,y\right)=2\theta \left(x,y\right)$ [38], [39], [40]. This phase modulation is crucial for the device’s ability to manipulate light polarization. Specifically, the phase modulations for LCP and RCP light are opposite, meaning $\varphi_{\text{LCP}}\left(x,y\right)=-\varphi_{\text{RCP}}\left(x,y\right)$ . This polarization-dependent phase modulation results in opposite focal lengths for LCP and RCP light. Considering the meta-device is ‘thin’ for simplicity, the combined focal lengths of the PB phase-based meta-lens, refractive lens, and ETL for LCP and RCP light are given by the following equations:

$$\begin{array}{c} \frac{1}{f_{\text{LCP}}}=\frac{1}{f_{\text{ME}}}+\frac{1}{f_{\text{RE}}}+\frac{1}{f_{\text{ETL}}},\\ \frac{1}{f_{\text{RCP}}}=-\frac{1}{f_{\text{ME}}}+\frac{1}{f_{\text{RE}}}+\frac{1}{f_{\text{ETL}}}. \end{array}$$

As shown in Figure 1(b), the phase distributions of the meta-lens for LCP and RCP are opposite, and the focal points of LCP and RCP are on different sides of the meta-lens. By being closely attached to a refractive lens with a much smaller focal length, the phase distribution can be changed to form two different focal points on one side. It should be noted that to give a better demonstration of the concept, the phase distributions in Figure 1(b) are magnified. The true phase distributions of the meta-device for LCP and RCP are close because the refractive lens dominates the primary phase distribution and the focal length. As shown in Figure 1(c), the meta-device produces two distinct focal points for LCP and RCP light. By positioning the polarized camera at the center between the two imaging planes, the camera can simultaneously record underfocused and overfocused images of the object. The image under LCP light is overfocused, and the image under RCP light is underfocused. These two defocused images captured by the polarized camera can be further processed for phase retrieval, a technique that extracts phase information from intensity measurements. The ETL can change the focal length to focus on objects of different distances. The theoretical derivation of how the polarization state of light changes and how the polarization camera can distinguish between LCP and RCP can be found in Supplementary Note 1.

**Figure 2:**
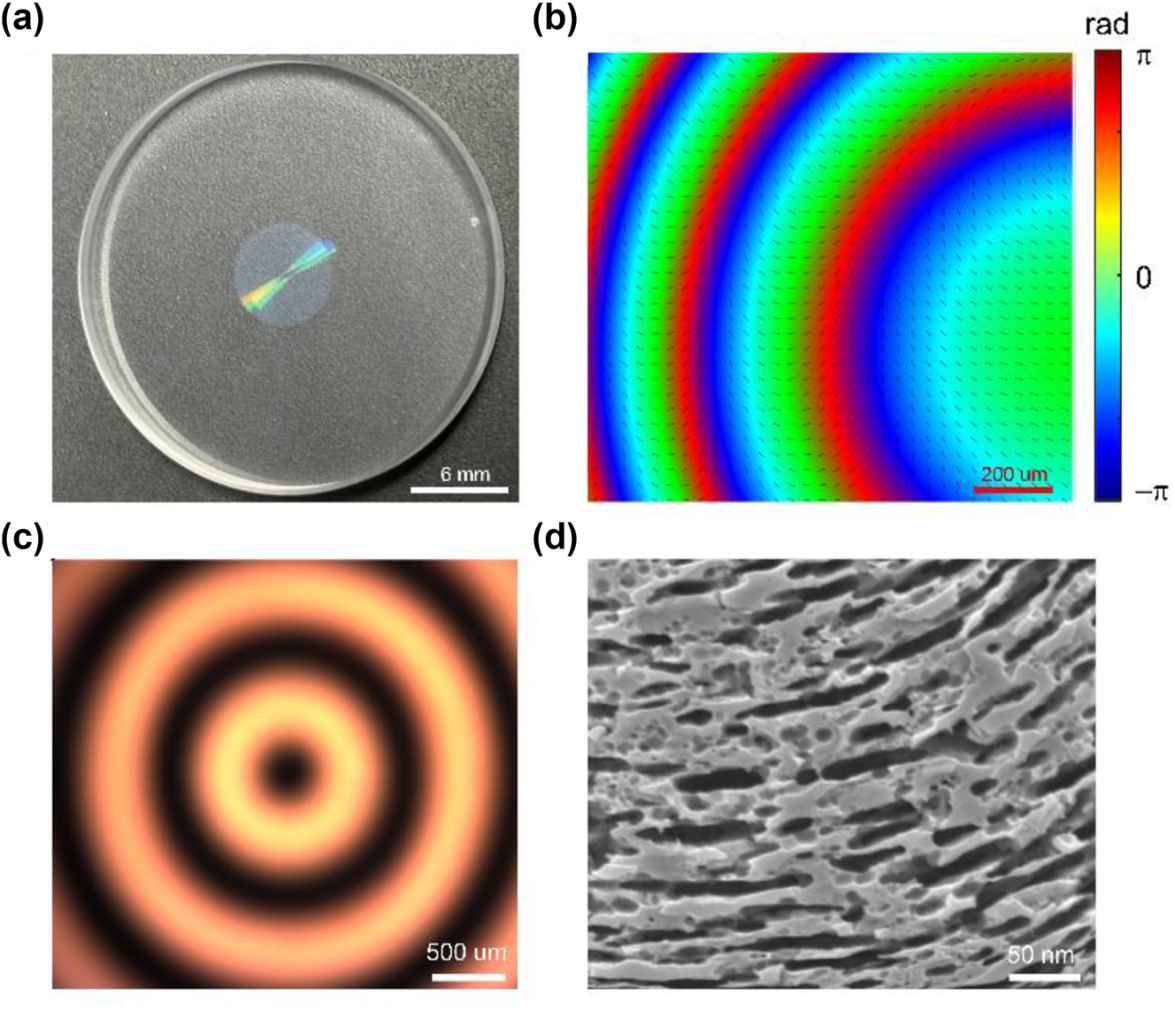
Characterization of the meta-lens. (a) Photograph of the meta-lens. (b) The zoomed-in experimental phase distribution of the meta-lens. (c) Crossed polarized image captured under light with 633 nm wavelength. (d) The zoomed-in SEM image of the meta-lens.

To demonstrate the concept, we designed a PB phase-based meta-lens with a 50 cm focal length and a 6 mm diameter, working at 633 nm of wavelength, created using form-birefringent as depicted in Figure 2(a). This meta-lens is fabricated through femtosecond laser writing, which introduces spatially varying nanostructures into fused silica glass. The resulting form-birefringence patterns are controlled by adjusting the writing parameters. Given that the nanostructures are at a deep subwavelength scale, they can be effectively treated as uniform media. At each point within the written area, the local optical axis aligns parallel and perpendicular to the nanostructures, effectively creating an artificial uniaxial crystal. The detailed fabrications are introduced in the Materials and Methods section. Figure 2(b) illustrates the local phase distribution of the meta-lens with incident LCP light captured in the experiment. The phase measurement method is described in Supplementary Note 2. Figure 2(c) displays the optical image when the meta-lens is between two crossed linear polarizers. A zoomed-in scanning electron microscope (SEM) image of the meta-lens is shown in Figure 2(d).

The TIE can be derived based on the paraxial approximation of monochromatic light [41],

$$-k\frac{\partial I\left(x,y\right)}{\partial z}=\nabla \cdot \left(I\left(x,y\right)\nabla \varphi \left(x,y\right)\right),$$

where the *k* is the wave vector, $I\left(x,y\right)$ and $\varphi \left(x,y\right)$ are the in-focus intensity profile and the phase profile, respectively. ∇is the gradient operator. With the help of an auxiliary function *ψ* satisfying∇*ψ* = *I*∇*φ* *,* the TIE can be converted to two Poisson equations [15]:

$$-k\frac{\partial I}{\partial z}=\nabla^{2}\psi ,$$

and

$$\nabla \cdot \left(I^{-1}\nabla \psi \right)=\nabla^{2}\varphi .$$

*I* is assumed to be constant for a pure phase object, and these two Poisson equations can be further simplified:

$$\begin{array}{c} -kI^{-1}\frac{\partial I}{\partial z}=\nabla^{2}\varphi ,\\ \varphi =-kI^{-1}\nabla^{-2}\frac{\partial I}{\partial z}, \end{array}$$

which can also be extended to a non-uniform intensity profile,

$$\varphi =-k\nabla^{-2}\nabla \cdot \left[I^{-1}\nabla \nabla^{-2}\frac{\partial I}{\partial z}\right],$$

which can be easily solved with the help of fast-Fourier transform (FFT), because ∇ and ∇^−2^ can be easily implemented in the Fourier domain.

**Figure 3:**
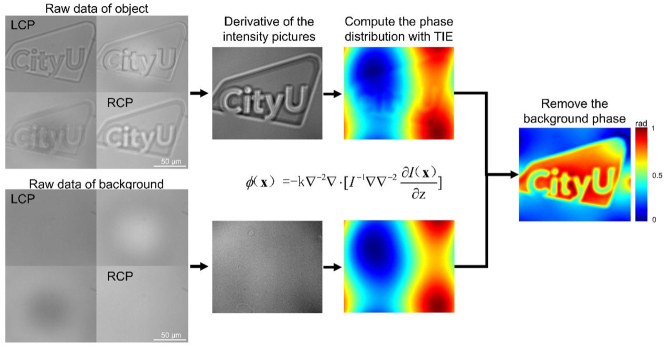
Flow chart of the phase imaging method. The LCP and RCP images are chosen from the raw data of the polarized camera for both object and background. A derivative of the intensity pictures using the two images. The phase distributions of objects can be derived by passing intensity pictures and the derivation into the TIE solution equation. The final phase distribution can be derived by removing the background phase.

Due to unavoidable experimental errors, such as the direction and uniformity of the incident light, as well as the thickness of the sample substrate, a background phase can be introduced. This background phase can potentially interfere with the phase information of the sample of interest, leading to inaccurate or misleading results. To mitigate this issue, the bare substrate area’s intensity information is imaged under the same experimental parameters to obtain the background phase. By subtracting this background phase from the phase data obtained with the sample present, the phase characteristics of the sample itself can be isolated. The overall algorithm flow, as depicted in Figure 3, initiates with acquiring raw data from the polarized camera for both the object of interest and the background substrate. This raw data includes the captured images under different linear polarization states. Specifically, the intensity images corresponding to RCP and LCP are extracted from these raw data sets. The difference between the RCP and LCP intensity images is calculated to obtain the intensity differential. This differential serves as a key input for the subsequent phase retrieval process. The intensity differential is then substituted into the TIE solution formula. However, the sample phase solution obtained directly from this process is usually superimposed on the phase provided by the substrate. To address this issue, the background phase, the phase distribution obtained from the substrate without the sample, is subtracted from the phase solution. This subtraction effectively removes the background noise, allowing for the accurate extraction of the phase information specific to the sample. By following this comprehensive approach, the algorithm ensures that the phase distribution of the sample is accurately analyzed, providing valuable insights into the sample’s properties.

**Figure 4:**
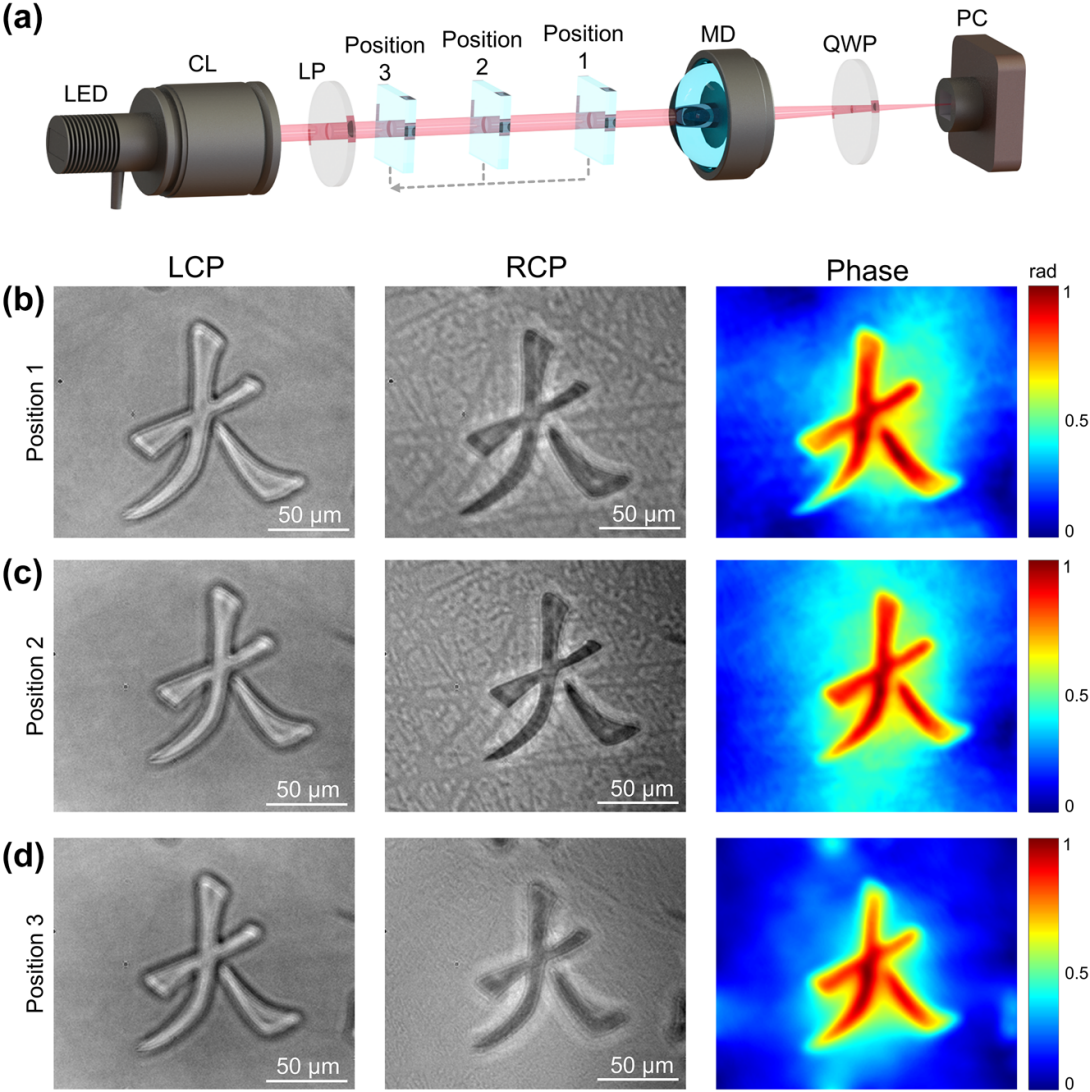
Experimental setup and the TIE phase retrieval results at different position (a) Experimental set up for three objects with different distances. CL: Condenser Lens; LP: Linear Polarizer; Position 1–3 is the object moved to the corresponding position; MD: Meta-device; QWP: Quarter-wave Plate; PC: Polarized Camera. (b,c,d) The object is moved to the position 1 to 3, respectively. The left column is the overfocused images with LCP images. The mid-column is the underfocused image with RCP. The right column is the phase image.

It should be noted that the working principle of the meta-device is employed by TIE, two images along the axial direction should be captured by the camera. Figure 4(a) shows the experiment setup of the tunable meta-device-based phase measurement system. When an LED source with a condenser lens and linear polarization illuminates the object, the output linear-polarized light with object information impacts the meta-device. Because of the working principle of the meta-device, the output light has dual focuses with two circle-polarizations (RCP and LCP). A quarter-wave plate is placed in front of the polarized camera to record the separated polarized images, which can be suitable for the TIE algorithm. Here, *d*_1_ and *d*_2_ stand for the object’s distance from the meta-device and the meta-device’s distance from the polarization camera, respectively. The relationship between *d*_1_ and *d*_2_ is $\frac{1}{d_{1}}+\frac{1}{d_{2}}=\frac{1}{f}$ . Because of the tunability of the meta-device’s focal length and the fixation of d_2_ in the experiment setup, d_1_ can be changed in order to adapt to different axial positions of the object. When the object moves to different positions, the depth of field of phase measurement is extended by electrically adjusting the focal length of the meta-device, which means that the depth of field of TIE can be extended by the proposed meta-device. The captured intensity images and phase retrieval results for objects with different distances are shown in Figure 4(b–d). The traditional TIE algorithms cannot perform quantitative phase imaging when the object is at different positions, our meta-device can still capture objects at various depths by adjusting the focal length. The total distance between position 1 and position 3 is 2.52 mm. The object used in the experiment demonstration is a pattern of a Chinese character ‘da’ made of 200 nm thick SiO_2_. The theoretical phase of this object is 0.91 radians. Comparing this with the phase recovered by our meta-device, the measured phase is about 0.914 radians, with a high accuracy of around 98.47 %. The accuracy of the TIE method can be demonstrated. A comprehensive characterization of the imaging performance of the meta-device at different positions can be found in Supplementary Note 3.

## 3 Discussion

In summary, we present a novel meta-device comprising a meta-lens, a refractive lens, and an ETL. This meta-device is designed to separate the images of LCP and RCP along the optical axis and to dynamically adjust the focal length, thereby enabling the capture of objects at varying distances without necessitating any mechanical movement of the overall optical system. By strategically positioning the polarized camera between two focal planes, acquiring both overfocus and underfocus images is feasible. The phase information can subsequently be derived using the TIE algorithm. The utilization of an LED light source in this study confers several distinct advantages over conventional quantitative phase imaging techniques based on interferometry. These benefits include enhanced resolution, diminished susceptibility to noise, and a more straightforward, cost-effective experimental setup. In contrast to traditional TIE quantitative phase imaging, which typically requires mechanical displacement to obtain different defocused images, our approach necessitates only a single capture, enhancing operational convenience. Conventional quantitative phase imaging methodologies are inherently limited in their ability to image objects at disparate depths. By contrast, through the modulation of focal length, our meta-device can effectively image objects at varying distances, thereby substantially augmenting the depth of field. Utilizing the FFT-based TIE algorithm in the methodology significantly enhances computational efficiency, enabling the calculation to be completed within 0.2 s. This rapid computational capability underscores the feasibility of real-time quantitative phase imaging of objects.

## 4 Materials and Methods

### 4.1 Meta-lens fabrication

The meta-lens substrate has a diameter of 25 mm and a thickness of 3 mm. The patterned area, positioned on a glass substrate, forms a circular region with a diameter of 6 mm. The fabrication process involves using a femtosecond laser to inscribe spatially varying nanostructures into a silica glass substrate via normal-incidence lasing writing. During the fabrication, the glass substrate is mounted on a computer-controlled rotating holder, allowing precise adjustment of the rotation speed. In addition, by gradually adjusting the polarization of the laser, nanostructures with continuously varying orientations can be fabricated. These self-organized nanostructures behave as form-birefringent materials, with their fast and slow axes aligned parallel and perpendicular to the stripes, respectively. The refractive index of the sample can be tuned by varying the laser irradiation intensity, resulting in birefringence within the initially isotropic glass substrate. This process causes the uniform silicon dioxide (SiO_2_) glass to decompose into porous silicon dioxide (SiO_2-x_). The refractive index of this porous glass is directly correlated with the laser intensity. The induced birefringent phase retardation, denoted as *ϑ*, is given by the equation: $\vartheta =2\pi \left(n_{e}-n_{o}\right)h/\lambda$ where *h* represents the writing depth, and $\left(n_{e}-n_{o}\right)$ signifies the induced birefringence. At a wavelength of 633 nm, the phase retardation *ϑ* of our sample is *π* achieved with a writing depth *h* of approximately 40 μm. The line width of the nanogrooves ranges from approximately 30 to 90 nm. Supplementary Note 4 shows more detailed information of the metalens’ design and fabrication.

### 4.2 Experiment

The polarized complementary metal–oxide semiconductor (CMOS) camera (BFS–U3–51S5P–C, IMX250MZR, FLIR) is used in the experiment, with a resolution of 2448 × 2048 for a total of four LP channels and a pixel size of 3.45 μm × 3.45 μm. An LED light (Thorlabs, M625L4-C1) provides partially incoherent light with fewer speckles. The ETL (Optotune, EL-10-30-C) is used to change the focal length of the meta-device. The refractive lens used in this work has a 25.4 mm diameter and a 25.4 mm focal length. By applying a current of 0–300 mA, a focal power range of 0–5 dpt, and a focal length goes from 200 mm to infinity.

## Supplementary Material

Supplementary Material Details
