# Supplementary material for: Tunable meta-device for large depth of field quantitative phase imaging
Source: Nanophotonics. 2025 Feb 17;14(8):1249–56. doi: 10.1515/nanoph-2024-0661 (PMC12019949; doi:10.1515/nanoph-2024-0661)
Supplement: Supplementary file 1 — Supplementary Material Details [file j_nanoph-2024-0661_suppl_001.pdf]

Supplement Information for

Tunable Meta-Device for Large Depth of Field Quantitative Phase Imaging

Jialuo Cheng[1,†], Zihan Geng[2,†], Yin Zhou[1,†], Zhendong Luo[1], Xiaoyuan Liu[1], Yinuo Xiang[1], Junxiao Zhou[1,*], and Mu Ku Chen[1,3,*]

[1]Department of Electrical Engineering, City University of Hong Kong, Kowloon, Hong Kong SAR 999077, China

[2]Institute of Data and Information, Tsinghua Shenzhen International Graduate School, Tsinghua University, Shenzhen, Guangdong 518071, China.

[3]State Key Laboratory of Terahertz and Millimeter Waves, City University of Hong Kong, Kowloon, Hong Kong SAR 999077, China

[†]These authors contributed equally to this work

*Correspondence: junxzhou@cityu.edu.hk ; mkchen@cityu.edu.hk

Supplementary Note 1: The theory of this work

Supplementary Note 2: Meta-lens sample phase distribution measurement

Supplementary Note 3: Characterization of the imaging performance of the meta-device at different positions

Supplementary Note 4: Meta-lens design and detailed information on the fabrication method

Supplementary Note 1: The theory of this work

The polarized camera used in this work has four linear polarization channels with different polarization angles of 0°, 45°, 90°, and 135°, which means it can't distinguish the LCP and RCP directly. For this requirement, for example, we can use a quarter-wave plate that changes LCP and RCP light to LP, such as 45° and 135°, respectively, which the polarized camera can characterize. The Jone matrix of meta-lens with a space rotation angle θ is $T_\theta = \begin{bmatrix} cos2\theta & sin2\theta \\ sin2\theta & -cos2\theta \end{bmatrix}$. When an x-polarized incident light, which can also be considered as two equal components of LCP and RCP light, passes through the meta-lens, the LCP will be changed to RCP, and RCP will be changed to LCP light, with opposite phase modulations.

$$\begin{bmatrix} cos2\theta & sin2\theta \\ sin2\theta & -cos2\theta \end{bmatrix}\begin{bmatrix} 1 \\ 0 \end{bmatrix} = \frac{1}{2}\begin{bmatrix} cos2\theta & sin2\theta \\ sin2\theta & -cos2\theta \end{bmatrix}\left(\begin{bmatrix} 1 \\ j \end{bmatrix} + \begin{bmatrix} 1 \\ -j \end{bmatrix}\right)$$

$$\text{RCP changed to LCP: } \frac{1}{2}\begin{bmatrix} cos2\theta & sin2\theta \\ sin2\theta & -cos2\theta \end{bmatrix}\begin{bmatrix} 1 \\ j \end{bmatrix} = \frac{e^{i2\theta}}{2}\begin{bmatrix} 1 \\ -j \end{bmatrix}$$

$$\text{LCP changed to RCP: } \frac{1}{2}\begin{bmatrix} cos2\theta & sin2\theta \\ sin2\theta & -cos2\theta \end{bmatrix}\begin{bmatrix} 1 \\ -j \end{bmatrix} = \frac{e^{-i2\theta}}{2}\begin{bmatrix} 1 \\ j \end{bmatrix}$$

After the light passes through the quarter-wave plate, whose fast axis is aligned with the x-axis, the LCP will be changed to 135° LP, and the RCP will be changed to 45° LP.

$$\text{LCP changed to 45° LP : } \frac{e^{i2\theta}}{2}\begin{bmatrix} 1 & 0 \\ 0 & j \end{bmatrix}\begin{bmatrix} 1 \\ -j \end{bmatrix} = \frac{e^{i2\theta}}{2}\begin{bmatrix} 1 \\ 1 \end{bmatrix}$$

$$\text{RCP changed to 135° LP : } \frac{e^{-i2\theta}}{2}\begin{bmatrix} 1 & 0 \\ 0 & j \end{bmatrix}\begin{bmatrix} 1 \\ j \end{bmatrix} = \frac{e^{-i2\theta}}{2}\begin{bmatrix} 1 \\ -1 \end{bmatrix}$$

When these two LP lights are captured by a polarized camera, they pass through four linear polarizations, and the corresponding responses are:

$$0° : \frac{e^{i2\theta}}{2}\begin{bmatrix} 1 & 0 \\ 0 & 0 \end{bmatrix}\begin{bmatrix} 1 \\ 1 \end{bmatrix} + \frac{e^{-i2\theta}}{2}\begin{bmatrix} 1 & 0 \\ 0 & 0 \end{bmatrix}\begin{bmatrix} 1 \\ -1 \end{bmatrix} = cos2\theta\begin{bmatrix} 1 \\ 0 \end{bmatrix}$$

$$45° : \frac{e^{i2\theta}}{2}\begin{bmatrix} \frac{1}{2} & \frac{1}{2} \\ \frac{1}{2} & \frac{1}{2} \end{bmatrix}\begin{bmatrix} 1 \\ 1 \end{bmatrix} + \frac{e^{-i2\theta}}{2}\begin{bmatrix} \frac{1}{2} & \frac{1}{2} \\ \frac{1}{2} & \frac{1}{2} \end{bmatrix}\begin{bmatrix} 1 \\ -1 \end{bmatrix} = \frac{e^{i2\theta}}{2}\begin{bmatrix} 1 \\ 1 \end{bmatrix}$$

$$90° : \frac{e^{i2\theta}}{2}\begin{bmatrix} 0 & 0 \\ 0 & 1 \end{bmatrix}\begin{bmatrix} 1 \\ 1 \end{bmatrix} + \frac{e^{-i2\theta}}{2}\begin{bmatrix} 0 & 0 \\ 0 & 1 \end{bmatrix}\begin{bmatrix} 1 \\ -1 \end{bmatrix} = sin2\theta\begin{bmatrix} 0 \\ 1 \end{bmatrix}$$

$$135° : \frac{e^{i2\theta}}{2}\begin{bmatrix} \frac{1}{2} & -\frac{1}{2} \\ -\frac{1}{2} & \frac{1}{2} \end{bmatrix}\begin{bmatrix} 1 \\ 1 \end{bmatrix} + \frac{e^{-i2\theta}}{2}\begin{bmatrix} \frac{1}{2} & -\frac{1}{2} \\ -\frac{1}{2} & \frac{1}{2} \end{bmatrix}\begin{bmatrix} 1 \\ -1 \end{bmatrix} = \frac{e^{-i2\theta}}{2}\begin{bmatrix} 1 \\ -1 \end{bmatrix}$$

The raw images in 45° and 135° channels have the same intensity profile as images under RCP and LCP incident light, respectively. At the same time, the pictures in 0° and 90° channels can be considered as the combination of the pictures under RCP and LCP incident light, which are useless in this work.

Supplementary Note 2: Meta-lens sample phase distribution measurement

The phase distribution measurement (Fig. 2(b) in the main text) is conducted using the PB phase element measurement method provided by equipment from MetronLens. The interferometric method for phase measurement was employed, and the optical setup is from our previously published work, Light Sci. Appl., 2021. 10. 52. One light beam passes through the meta-lens, while the other is a reference beam. The two beams are combined before the CCD to generate interference fringes. By performing a Fourier transform on the captured interference pattern, the first-order information is extracted and subjected to an inverse Fourier transform. The resulting data is then divided by the interference pattern obtained without the meta-lens to derive the phase information of the meta-lens. To be more detailed, the formula is presented. When there are no samples in the object's optical path. We can write the complex amplitude of the object beam as

$$\widetilde{U}_1 = A_1 \exp[i(\boldsymbol{k}_1 \cdot \boldsymbol{r} + k_{1z} z_1 + \psi_1(x,y))]$$

where $\boldsymbol{k}_1 = (k_{1x},\ k_{1y})$, $\boldsymbol{r} = (x,y)$, k_{1z} is the z component of the wave vector, $\psi_1(x,y)$, is phase distribution of the object beam.

Put the metalens in the optical path of the object beam, leading to

$$\widetilde{U}_2 = A_2 \exp[i(\boldsymbol{k}_1 \cdot \boldsymbol{r} + k_{1z} z_1 + \psi_1(x,y) + \varphi_{metalens}(x,y))]$$

, where $\varphi_{metalens}(x,y)$ is phase distribution of the metalens.

For the reference beam, the complex amplitude is given by

$$\widetilde{U}_3 = A_3 \exp[i(\boldsymbol{k}_3 \cdot \boldsymbol{r} + k_{3z} z_3 + \psi_3(x,y))]$$

, where $\boldsymbol{k}_3 = (k_{3x},\ k_{3y})$, $\psi_3(x,y)$ is the phase distribution of the optical path of the reference beam.

Then the interference fringe intensity pattern was produced at the CCD, namely,

$$I(x,y) = (\widetilde{U}_2 + \widetilde{U}_3)^* \cdot (\widetilde{U}_2 + \widetilde{U}_3) = |A_2|^2 + |A_3|^2 + A_2^* A_3 \exp\left[i\left((\boldsymbol{k}_3 - \boldsymbol{k}_1) \cdot \boldsymbol{r} + k_{3z} z_3 - k_{1z} z_1 + \psi_3(x,y) - \psi_1(x,y) - \varphi_{metalens}(x,y)\right)\right] + A_3^* A_2 \exp\left[-i\left((\boldsymbol{k}_3 - \boldsymbol{k}_1) \cdot \boldsymbol{r} + k_{3z} z_3 - k_{1z} z_1 + \psi_3(x,y) - \psi_1(x,y) - \varphi_{metalens}(x,y)\right)\right]$$

By operating Fourier-transformation on the fringe pattern, we can get

$$\tilde{I}(k_x, k_y) = \tilde{I}(0) + \tilde{I}(\Delta k) + \tilde{I}(-\Delta k)$$

, where $\Delta k = \boldsymbol{k}_3 - \boldsymbol{k}_1$. We retain the term of

$$\tilde{I}(\Delta k) = A_2^* A_3 \exp\left[i\left((\boldsymbol{k}_3 - \boldsymbol{k}_1) \cdot \boldsymbol{r} + k_{3z} z_3 - k_{1z} z_1 + \psi_3(x,y) - \psi_1(x,y) - \varphi_{metalens}(x,y)\right)\right]$$

Then by inverse Fourier transformation and taking its argument, the difference of phase

distribution can get,

$$\varphi_3 - \varphi_2 = (\boldsymbol{k}_3 - \boldsymbol{k}_1) \cdot \boldsymbol{r} + k_{3z}z_3 - k_{1z}z_1 + \psi_3(x,y) - \psi_1(x,y) - \varphi_{metalens}(x,y)$$

Remove metalens out of the object beam， we can easily obtain the phase contributions from the measurement system,

$$\varphi_3 - \varphi_1 = (\boldsymbol{k}_3 - \boldsymbol{k}_1) \cdot \boldsymbol{r} + k_{3z}z_3 - k_{1z}z_1 + \psi_3(x,y) - \psi_1(x,y)$$

Combining the above two equations， the phase distribution of metalens $\varphi_{metalens}(x,y)$ can be obtained.

Supplementary Note 3: Characterization of the Imaging Performance of the meta-device at different positions

To characterize the imaging performance of the meta-device, we have supplemented the experimental results of the quantitative phase imaging system at different positions. The object distance discrepancy between the Left Circularly Polarized (LCP) and Right Circularly Polarized (RCP) channels is measured when the electronically tunable lens (ETL) is positioned at various optical powers. Additionally, the field of view and spatial resolution of both the LCP and RCP channels are quantified. Our algorithm employs the Transport of Intensity Equation (TIE), which reconstructs the phase image from two defocused intensity images. Consequently, the spatial resolution of the amplitude and phase images should be identical, and thus, we did not specifically differentiate between the spatial resolution of the amplitude and phase. The larger the optical power value, the smaller the focal length of the ETL, and, consequently, the smaller the overall focal length of the meta-device. As can be observed from Table S1, with the increase in optical power, there is a slight reduction in defocusing distances, and both the imaging magnification and field of view of the system undergo minor changes, albeit not significantly. The resolution identified by the Rayleigh criterion remains consistent across different positions at 4.92 µm in linewidth (group 6, element 5). Images at various distances are presented in Figure S1 with the values of the average of dips divided by the average of peaks are shown on the top right corner. The values are all smaller than the Rayleigh criterion 0.8114, which means that group 6 element 5 can be seen.

Table S1. Image performance of the meta-device at different positions. OP: Optical power range; DD: Defocusing distances; IM: Imaging magnification; FOV: Field of view; RE: Resolution;

OP (dpt)	DD (mm)	LCP			RCP		
		IM	FOV (um)	RE (um)	IM	FOV (um)	RE (um)
1	3.04	14.18	297.87	4.92	15.27	276.59	4.92
2	2.85	14.38	293.76	4.92	15.66	269.59	4.92
3	2.8	14.57	289.76	4.92	15.76	267.89	4.92
4	2.71	14.77	285.87	4.92	15.86	266.22	4.92
5	2.61	14.97	282.087	4.92	16.06	262.93	4.92

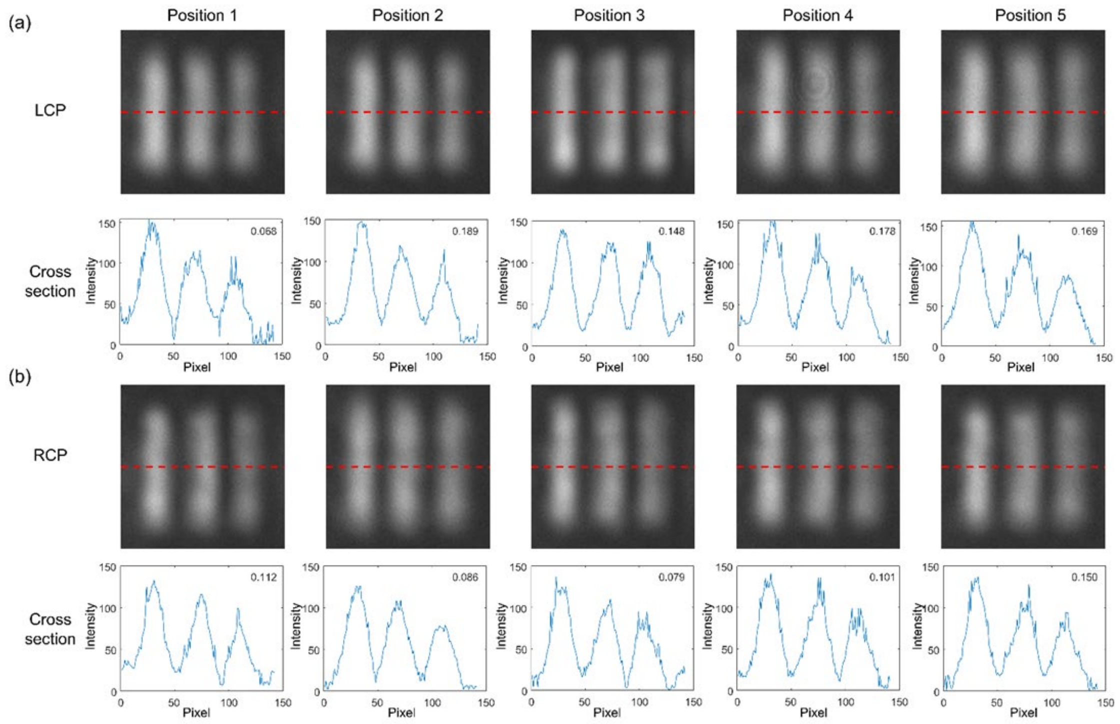

Figure S1. LCP and RCP images of group 6 element 5 of 1951 USAF resolution test chart and cross sections by the meta-device.

Supplementary Note 4: Meta-lens design and detailed information on the fabrication method

The phase distribution of the metalens could be expressed as: $\varphi(x,y) = -\frac{2\pi}{\lambda}(\sqrt{x^2+y^2+f^2}-f)$. Here, $\lambda = 532\,nm$ is the wavelength, and $f = 50\,cm$. As shown in our SEM images, the meta-structures have a nano-groove shape. The width of the nano-groove is around 30 nm. The period of the nano-groove is around 300 nm. The key design parameters of the metalens are determined by the fabrication method. The fabrication details, followed by the design specifics, are outlined below:

Our sample is fabricated using laser writing technology in transparent SiO_2. Under intense laser irradiation, multiphoton ionization generates a high-density free electron plasma, imparting plasma-like properties to the SiO_2. The plasma wave interferes with the incident laser beam, forming stripe-like nanoscale grooves (approximately 30 nm in width). These stripes are oriented perpendicular to the polarization direction of the writing laser. By gradually varying the polarization of the writing laser, nanostructures with continuously changing orientations can be obtained. Simultaneously, under the intense laser illumination, the uniform SiO_2 sample decomposes into porous glass (SiO_{2-x}), forming birefringent materials, with the fast and slow optical axes aligned parallel and perpendicular to the strip, respectively.

The effective ordinary and extraordinary refractive indices are given by $n_o = \sqrt{\text{Ƒ}n_1^2+(1-\text{Ƒ})n_2^2}$, $n_e = \sqrt{n_1^2 n_2^2/[\text{Ƒ}n_1^2+(1-\text{Ƒ})]n_2^2}$, where n_1 and n_2 are the refractive indices of the two media forming the nanostructure, and Ƒ is the filling factor. Designing a phase retardation of π is crucial for optimal optical effects and efficient conversion between linear and circular polarization. The phase retardation is given by $\vartheta = \frac{2\pi h}{\lambda}(n_e - n_o)$, where h represents the writing depth and $n_e - n_o$ represents the induced birefringence. By selecting appropriate values for the filling factor Ƒ and the thickness h, the desired phase retardation, akin to that of conventional birefringent crystals such as liquid crystals, can be achieved. In this study, we selected a phase retardation $\vartheta=\pi$, with h = 40 μm and a filling factor of approximately 0.2, based on the induced birefringence at the 10^{-3} level.
